# Formal Semantics in the Neurology Clinic: Atypical Understanding of Aspectual Coercion in ALS Patients

**DOI:** 10.3389/fpsyg.2016.01733

**Published:** 2016-11-04

**Authors:** Giosuè Baggio, Giulia Granello, Lorenzo Verriello, Roberto Eleopra

**Affiliations:** ^1^Language Acquisition and Language Processing Lab, Norwegian University of Science and TechnologyTrondheim, Norway; ^2^Neurology Unit, Azienda Ospedaliero Universitaria Ospedali RiunitiTrieste, Italy; ^3^Neurology Unit, Azienda Ospedaliero Universitaria Santa Maria della MisericordiaUdine, Italy

**Keywords:** amyotrophic lateral sclerosis, semantics, aspectual coercion

## Abstract

Amyotrophic lateral sclerosis (ALS) is a neurodegenerative disease of the motor system with subtle adverse effects on cognition. It is still unclear whether ALS also affects language and semantics, and if so, what aspects and processes exactly. We investigated how ALS patients understand verb phrases modified by temporal preposition phrases, e.g., “To watch TV for half an hour.” Interpretation here requires operations such as *aspectual coercion* that add or delete elements from event structures, depending on temporal modifiers, and *constraints on coercion*, which make combinations with certain modifiers not viable. Using a theoretically-motivated experimental design, we observed that acceptance rates for aspectual coercion were abnormally high in ALS patients. The effect was largest for the more complex cases of coercion: not those that involve *enrichment* of event structures (“To switch on the TV in half an hour,” where a number of failed attempts must be included in the interpretation) but those that, if applied, would result in *deletion* of event structure elements (“To repair the TV for half an hour”). Our experimental results are consistent with a deficit of constraints on coercion, and not with impaired semantic processes or representations, in line with recent studies suggesting that verb semantics is largely spared in ALS.

## Introduction

Amyotrophic lateral sclerosis (ALS), or Lou Gehrig's disease, as is also known in some countries, is among the most common neurodegenerative conditions in humans. It predominantly affects the motor system, though behavioral and cognitive effects with varying degrees of severity are documented in 25–50% of patients (Phukan et al., 2007, 2011; Consonni et al., 2013; Goldstein and Abrahams, 2013). The hypothesis of a continuum between ALS and frontotemporal dementia (FTD) has recently acquired increasing support (Strong et al., 2003; Mackenzie, 2007; Turner et al., 2013): 5–15% of ALS patients are diagnosed with frontotemporal dementia (FTD), and 10% of FTD patients develop ALS. About 5–7% of all ALS cases are familial with a Mendelian inheritance pattern whereas the remaining 93–95% are classified as sporadic. There exist several variants of ALS. Some have been linked to gene mutations which account for a share of both familial (23% by C9orf72 mutations, 4% by FUS/TLS, 20% by SOD1 and 5% by TDP-43) and sporadic (5–7% by C9orf72, 1% by SOD1 and 2% by TDP-43) variants of ALS (Turner et al., 2013). The genetic bases of ALS are increasingly well understood, although identifying susceptibility genes of the sporadic forms has proved challenging. There are, on the other hand, few known environmental variables that determine ALS risk factors: athletes and manual workers seem to be especially vulnerable social categories. The main presentations of the disease are limb-onset and bulbar-onset ALS. Both may result in spastic dysarthria (with slow, labored and distorted speech) and in cognitive decline (Hardiman et al., 2011; Kiernan et al., 2011; Silani et al., 2011; Turner et al., 2013). The ALS phenotype reflects breakdown of a broad motor network, and its manifestations across the nervous system are often diverse. The brain of ALS patients, in particular, shows thinning of bilateral precentral gyri (primary motor cortex), occasionally extending to somatosensory areas, and to a lesser extent to frontal and temporal areas (Tsermentseli et al., 2012; Verstraete et al., 2012). Decreases in fractional anisotropy (an index of white matter integrity) have been found in the corpus callosum and in white matter fibers underlying (pre)motor regions (Filippini et al., 2010; Cirillo et al., 2012).

ALS has been discussed and used as a model for exploring functional relations between motor systems (and their impairment) and representation and processing at different levels of the organization of the language system, including semantics. Several studies on verb production and comprehension in ALS have been published (see below). There is much less research on more complex yet equally important structures: verb phrases, modifiers, sentences and discourse. The study of neurological patients, both in basic and in clinical research, routinely relies on standardized tests, which may be combined with additional ad-hoc tests. Here we argue that there is considerable potential for augmenting current neuropsychological batteries, in the study of aphasia and language disorders in general, with special-purpose tests based on semantic theory. There is room for exploring the status of a wide range of operations beyond single-words in neurological conditions, that may shed light both on the cognitive profiles of such conditions and on the components (dissociable or otherwise) that contribute to core operations in language.

The neurological condition investigated here is ALS, and the linguistic operation in focus is aspectual coercion. Aspectual coercion can informally be defined as a transformation of the default meaning of verb phrases, triggered by a modifier phrase. For example, a semantic representation in which a goal state is implied (“She wrote the letter in 2 hours,” suggesting she *finished* the letter in 2 hours) may be turned, via aspectual coercion, into a new structure where the goal state is missing (“She wrote the letter for 2 hours,” suggesting she worked on a draft for 2 hours, without completing the letter). Aspectual coercion is triggered by specific temporal modifiers (e.g., “for 2 hours”) being applied to verb phrases. Aspectual coercion is well-understood theoretically and experimentally, which makes it an optimal candidate for use in a clinical context. Furthermore, studying aspectual coercion in ALS patients allows us to fill an important gap in the literature, by providing novel data on language processing beyond single words in the domain of action and event semantics in ALS. In particular, we will test three (alternative) hypotheses: ALS patients exhibit comprehension deficits either (i) in the representation of verb phrase semantics, or (ii) in aspectual coercion operations, or (iii) in the semantic and pragmatic constraints that govern the application of such operations. Before we formulate these hypotheses more precisely, we will briefly review earlier research on language and cognition in ALS.

### Language and cognition in ALS

Previous research has described a range of cognitive deficits in ALS patients, including reduced verbal fluency, impaired attention and working memory in immediate recall and digit-span tasks, semantic paraphasias (substitutions of words with semantically related words in speech production), difficulties in object naming and syntactic comprehension, and perseverations, though not visual-perceptual dysfunction (e.g., in navigation; for reviews see Phukan et al., 2007; Goldstein and Abrahams, 2013). However, in ALS patients motor confounds are often difficult to discount. For example, verbal fluency is under executive control in healthy adults, but is often impaired in ALS patients. Yet, that is a possible direct effect of ALS on motor effectors and muscles, leading to dysarthria (not necessarily dysphasia or aphasia), rather than an indirect effect on executive systems in the brain. A recent meta-analysis of 16 studies with a total of 554 non-demented ALS patients takes into account diminished motor speed, in speech production too, in interpreting meta-data (Raaphorst et al., 2010). The results point to a small set of cognitive-behavioral functions where ALS patients show poorer performance. Moreover, previous findings, e.g., reduced psychomotor speed, language and executive dysfunction in ALS, are likely to be explained by publication biases (Raaphorst et al., 2010), based on fail-safe *N* calculations (Rosenthal, 1979). A study on a large cohort of ALS patients (160), tested within 12 months of diagnosis on a neuropsychological battery of executive, memory, language and visuospatial tasks, reported that 14% of patients fulfilled the criteria for frontotemporal dementia (FTD), but as much as 50% had no cognitive deficits (Phukan et al., 2011). Impairment of language, memory and executive functions tended to co-occur in a fraction of non-demented ALS patients. One study of non-demented ALS patients found evidence for action naming difficulties, memory and executive dysfunction in about 30% of patients (Consonni et al., 2013). Recent work has described an association in about 40% of ALS patients between language impairment (in spelling, naming, synonymy, and grammar tasks; for recent results on syntax, see Tsermentseli et al., 2016) and executive dysfunction (Taylor et al., 2013).

Abrahams et al. (2000) investigated, controlling for motor speed, the sources of deficits of verbal fluency in ALS. Phonological loop functions were found to be largely intact, but ALS patients showed reduced working memory and difficulties in speech production (e.g., in generating animal names), with spared single word retrieval in a sentence completion task. Verbal dysfluency in ALS is likely a consequence of executive dysfunction, affecting supervisory attentional and central working memory systems, and not of phonological or linguistic impairment. Other work suggests that language can be disrupted in ALS. Bak and Hodges (1997) described three ALS patients with aphasia and FTD, who showed poorer performance in a word-picture matching task with verbs than nouns. Bak et al. (2001) describe six ALS patients who developed a progressive non-fluent aphasia with impaired syntactic comprehension and dementia, who had more difficulties in naming actions than objects shown in drawings, and matching a spoken verb with an appropriate action, compared to matching a spoken noun with an object. In these ALS patients, pathological changes (atrophy and gliosis) were found in BA44/45. Reduced activations in fluency and naming tasks in middle and inferior frontal gyri have been found using fMRI in ALS (Abrahams et al., 2004). Bak and Hodges (2004) tested three ALS patients with dementia and aphasia in a picture-picture matching task involving objects or actions. Patients performed worse with verbs than with nouns. It has been argued that verb impairment in ALS results from damage to the motor system, which appears necessary to instantiate aspects of action and verb semantics (Bak and Chandran, 2012). Recent work has challenged this conclusion. Papeo et al. (2015b) assessed ALS patients for comprehension of verbs and nouns related to the same motor representations (e.g., “write” and “pen”). Verbs resulted in more errors than nouns, but the effect was similar in ALS patients and controls. Patients showed impaired performance in action sequencing, consistent with previous findings of deficits of action knowledge in ALS (Grossman et al., 2008). This dissociation between action organization and action verb semantics in ALS suggests that the relative difficulty of verbs in patients and controls is not due to damaged motor representations, but to other morphosyntactic or semantic properties of verbs, that account for their complexity relative to nouns.

Importantly, most studies of language processing in ALS patients use *single-word* production or comprehension tasks, with few notable exceptions. Some studies have reported degraded sentence expression, in quantity, rates and errors, in speech production and grammar (Ash et al., 2015; Tsermentseli et al., 2015, 2016). Recent research has also provided evidence for deficits in the construction of narratives and other discourse-level or pragmatic aspects of language in ALS (Staios et al., 2013; Bambini et al., 2016). For example, Ash et al. (2014) used the children book “Frog where are you?,” a collection of 24 detailed drawings to be ordered in a story-like sequence, depicting numerous failed attempts made by a boy to find his pet frog that has escaped from a jar. Typically, participants are asked to narrate the story describing each picture in a sequence. ALS patients' narrations were scored for local connectedness (each event is linked with the previous one using adverbs, pronouns, definite noun phrases, cause and effect expressions etc.), global connectedness (here a single variable: whether the frog that is sought at the beginning of the story is the same frog that is found at the end), and maintenance of the story theme (how frequently the search theme is mentioned). ALS patients scored poorly in local connectedness and theme maintenance, regardless of whether there was any concomitant executive dysfunction and FTD. Gray matter atrophy in right dorsolateral prefrontal and bilateral inferior frontal cortices correlated with poor local connectedness. Ash et al. (2014) is one of the few studies that address semantic processing in ALS beyond single-words, however its results are more relevant to executive dysfunction in ALS than to language disorder: they indicate an impairment in action sequencing that is consistent with the findings of Papeo et al. (2015b). To our knowledge, no one has investigated intermediate levels of semantic representation (phrases or sentences) in ALS patients. We aim to fill this gap, using verb phrases (VPs: e.g., “to watch TV”) combined with different temporal modifiers, in preferred (e.g., “to watch TV for half an hour”) and non-preferred combinations (“to watch TV in half an hour”). A non-preferred combination is not necessarily anomalous, as it may result in a coherent representation if the meaning of the VP is transformed so as to suit the requirements of the given modifier phrase. In formal semantics, these transformations are called *aspectual coercions*. In what follows, we first briefly introduce the formal theory of event structure and aspectual coercion, and we then describe our own experimental design and hypotheses.

### Aspectual coercion

Theories of verb and VP semantics posit three formal components that play a role in the interpretation of sentences and discourses. The first component is the meaning of the VP, or *event structure* (Section Event structure), which specifies the temporal and causal profiles of the event denoted by the VP, e.g., whether it features a preparatory phase, a culmination and a goal state etc. The second component is a set of operations, including *aspectual coercion* (Section Aspectual Coercion), that transform the event structure that is associated by default to a VPs depending on temporal modifiers and other expressions in discourse. The third component is a set of *constraints* on coercion operations (Section Constraints on Coercion), requiring that the event structures that result from the transformation are consistent with lexical meanings and event knowledge. Below we discuss each of these components in turn.

#### Event structure

In theories of tense, aspect and event structure, the unit of analysis is the verb phrase or VP, and not the verb. VPs are classified into 5 main aspectual classes (or *Aktionsarten*), depending on the *internal structure* of the event they denote: *activities* (“to run,” “to push a cart”), *accomplishments* (“to cross a street,” “to write a letter”), *achievements* (“to arrive,” “to reach the top”), *points* (“to flash,” “to hop”), and *states* (“to know,” “to love”) (Vendler, 1957, 1967; Moens and Steedman, 1988; van Lambalgen and Hamm, 2004). The event structure underlying each aspectual class can be derived by combining 4 basic event elements: a *process* or activity (***p***; e.g., writing), which exerts a force on an object or *incremental theme* (***i***; e.g., the amount of letter written), a *culmination* (***e***; e.g., the completion of the letter), and a *goal* or consequent state (***g***; e.g., a complete letter). These elements may be either present (“+”) or absent (“−”) in event structures (“^*^” indicates an optional element):

$$\begin{array}{l} \;\begin{array}{llll} p & \;i & \;c & \;g \end{array}\\ \begin{array}{lllllll} \text{Activity} & ( & + & {+}^{*} & - & - & )\\ \text{Accomplishment} & ( & + & + & + & + & )\\ \text{Achievement} & ( & - & - & + & + & )\\ \text{Point} & ( & - & - & + & - & )\\ \text{State} & ( & - & - & - & + & ) \end{array} \end{array}$$

There is a *defeasible* link between a VP and its event structure, which can be overridden by context. Alternatively, one can model these flexible mappings as probability distributions over VPs for each aspectual class (Pulman, 1997).

#### Aspectual coercion

The default or preferred assignment of VPs to event structures can be modified by a variety of operations (see Dölling, 2014 for an extensive list) referred to as *coercion*. In a compositional interpretation of the sentence (van Lambalgen and Hamm, 2004, p. 166):

                   “Pollini played the sonata for 2 days.”

there is a semantic mismatch between knowledge of the duration of a typical sonata (even the longest ever written) and the time interval specified by the temporal modifier “for 2 days” (a prepositional phrase, or PP). It is possible to compute a viable interpretation here, where Pollini plays the same sonata *repeatedly* over 2 days. (The alternative reading in which Pollini played the sonata once, but very slowly for 2 days, seems less plausible, for it conflicts with knowledge of how such music is normally played.) The accomplishment “to play the sonata,” featuring a *single* culmination and a goal state, is coerced into an activity, removing the ***c*** and ***g*** elements from the event structure. The completion of a sonata (a single execution) is part of the activity, and may be iterated without a primary goal. More generally, one may turn an activity (“to write”) into an accomplishment (“to write a letter”) by adding an incremental theme (“a letter”) and its culmination point. That is a case of *additive coercion*: by composing a VP with a direct object, the event structure is *expanded* with ***i*** (the amount of letter written), ***c*** (the stage of completion of the letter), and ***g*** (a complete letter) elements (“↝” denotes coercion):

ACT(+−−−) ↝ACC(++++)

Conversely, one may turn an accomplishment (“to drink a glass of wine”) into an activity (“to drink wine”) by means of syntactic modification of the object NP—semantically, via *subtractive coercion*, where the ***i*** (the amount of wine drunk), ***c*** (drinking the last drop of wine in the glass), and ***g*** (an empty glass) elements are *deleted* from the event structure:

ACC(++++) ↝ACT(+−−−)

Other forms of aspectual coercion such as *cross coercion* are possible (see Section The Hypothesis Space, and van Lambalgen and Hamm, 2004). Besides adding an NP complement (e.g., “to write”↝“to write a letter”) and modifying an existing one (“to drink a glass of wine”↝“to drink wine”), aspectual coercion can also be triggered by adding PPs, such as the temporal modifiers introduced by the prepositions “in,” “for,” “at,” and others (De Swart, 1998).

Activities (ACT), achievements (ACH) and accomplishments (ACC) can in principle be coerced (“↝”) into each other, giving rise to 6 sentences types involving coercion, and 3 preferential (“no coercion”) VP+PP combinations. In our study, conducted in Italian, we used all 9 sentence types (shown in Table 1). It is in principle possible to construct for each of these 9 sentence types an interpretation preventing a semantic mismatch. That is more or less difficult to achieve, however, depending on the particular combination of a VP and PP in each construction. In some cases, that requires disregarding a conflict with event knowledge (e.g., the duration of a sonata, recall the example above) or with “lexical knowledge,” stretching the meaning of a VP beyond its “breaking point.” Aspectual coercion is the family of operations that must be invoked to derive such alternative interpretations. The easiest cases are those where the derivation can follow a strictly compositional logic, i.e., (1), (6), (8) (Table 1). These sentences do not involve aspectual coercion, so speakers are expected to accept them as grammatical and meaningful.

**Table 1:** **Examples of stimulus sentences and relative coercion operations**.

**Sample sentence**	**Condition**	**Control: M (SD)**	**ALS: M (SD)**
(1) Guardare la televisione per mezz'ora.	(ACT↝ACT; no coercion)	11.38 (3.78)	13.85 (1.99)
*To watch the television for half an hour*.			
(2) Guardare la televisione in mezz'ora.	(ACT↝ACC; additive coercion)	8.54 (4.2)	13.08 (3.59)
*To watch the television in half an hour*.			
(3) Guardare la televisione a mezzogiorno.	(ACT↝ACH; cross coercion)	12 (3.37)	14.46 (1.81)
*To watch the television at noon*.			
(4) Accendere la televisione per mezz'ora.	(ACH↝ACT; cross coercion)	7.54 (3.43)	10.77 (2.28)
*To switch on the television for half an hour*.			
(5) Accendere la televisione in mezz'ora.	(ACH↝ACC; additive coercion)	7.08 (3.66)	10 (2.61)
*To switch on the television in half an hour*.			
(6) Accendere la televisione a mezzogiorno.	(ACH↝ACH; no coercion)	12.62 (3.93)	14.77 (1.88)
*To switch on the television at noon*.			
(7) Aggiustare la televisione per mezz'ora.	(ACC↝ACT; subtractive coercion)	9.15 (3.63)	12.69 (3.17)
*To repair the television for half an hour*.			
(8) Aggiustare la televisione in mezz'ora.	(ACC↝ACC; no coercion)	10.85 (4.36)	13.69 (2.93)
*To repair the television in half an hour*.			
(9) Aggiustare la televisione a mezzogiorno.	(ACC↝ACH; subtractive coercion)	10.38 (4.29)	14.23 (1.83)
*To repair the television at noon*.			

*Mean acceptance rates (and standard deviations) for each sentence type in ALS patients and controls are also shown. These data are summarized and analyzed by condition in Table 4, and comparing all conditions to the no-coercion condition in Table 5. Values are rounded to the second decimal figure*.

In all the other cases, the combination of VP and modifier introduces a *semantic mismatch* between the event structure (provided by the VP) and the temporal frame in which it is situated (specified by the PP). To defuse these mismatches, aspectual coercion can be triggered to adjust the event structure either by adding a process, or a consequent and goal state (*additive coercion*), or by deleting a process or a consequent and goal state (*subtractive coercion*), or a combination of these (*cross coercion*). Whether coercion *may* be applied, and whether it *does* result in a possible interpretation for a given sentence, is a non-trivial matter which depends upon the semantics of the VP and PP, and on knowledge associated with them. Sentences (2)–(5), (7), and (9) should be more difficult to interpret for speakers: we therefore expect them to *reject* at least a fraction of these constructions.

#### Constraints on coercion

Constraints on coercion are needed to prevent one's theory from over-generating meanings (Pustejovsky and Bouillon, 1995; Pylkkänen, 2008). Dispreferred interpretations may be either *blocked* before semantic operations (e.g., aspectual coercions) are applied, or *discarded* after application. On the latter view, compositional processes would *supply* a set of interpretations. Contextual reasoning, based on lexical and event knowledge, would either *select* among compositionally-generated alternatives, or suggest a preference ranking over that set (Pulman, 1997; Dölling, 2014). Either way, interpretation involves two steps. First, a class of compositional meanings is generated. Second, *world knowledge*, e.g., knowledge of the *typical duration* of events is used to select the best interpretation, given a context. Some of the compositional structures generated during the first step contain parameters and variables that must be assigned to specific values via forms of reasoning based on event knowledge, during the second step (Dölling, 2014; see Baggio et al., 2010 for a similar proposal in the context of complement coercion, and Hagoort et al., 2009; Baggio et al., 2012a,b, 2015, 2016 for discussions of unification in semantics). On this view, some of the interpretations generated compositionally are underspecified, and full(er) semantic specification arises precisely from the (constrained) application of coercion and related semantic operations.

Lexical and event knowledge are not the only source of constraints on aspectual coercion. Koontz-Garboden (2007) introduces *monotonicity* as one such constraint: elements may be added to an event structure, but cannot be removed. The prediction here is that both subtractive and cross coercion are often less acceptable, and in the limit never acceptable, compared to additive coercion. Michaelis (2003, 2004) suggests a related, albeit weaker constraint, namely *Aktionsart preservation*: in aspectual coercion input and output event structures should share some elements. In this analysis, cross coercion would be usually disallowed, but Michaelis introduces the notion of a *chain mapping* with an intermediate step: e.g., turning activities into achievements requires that the event structure is transformed into an intermediate representation (i.e., an accomplishment), sharing the first two elements with activities, and the last two with achievements. This account still predicts that cross coercion is more complex because of the chain mapping it involves.

### The hypothesis space

A growing body of experimental research is employing behavioral and neural dependent variables to investigate the processing costs of aspectual coercion. Piñango et al. (1999) were the first to show, using a lexical decision task, that responses took longer when sentences required aspectual coercion (iterating a punctual event: e.g., “The insect hopped effortlessly until it reached the far end of the garden” vs. “The insect glided effortlessly until it reached the far end of the garden”; see also Todorova et al., 2000; Piñango et al., 2006). Evidence for delayed aspectual processing was provided by Piñango et al. (2006), who showed experimentally that the costs of coercion are not present at the point of syntactic licensing (e.g., “until” in the examples above), but appear further downstream in the sentence (Bott, 2010; Bott and Hamm, 2014; Bott and Gattnar, 2015). Although, subsequent research found no effects in self-paced reading and eye-tracking experiments for iterative coercions (“hopped”) (Pickering et al., 2006), Townsend (2013) has recently reported increased eye fixations in the adverb and post-adverb regions in iterative coercions, such as in “Howard sent a large check to his daughter for many years.” Piñango and Zurif (2001) investigated jointly complement coercions (“The boy began the book,” where an activity, such as reading, is included in the VP's event structure), aspectual coercions (“The girl jumped until dawn,” requiring iteration) and transparent sentences (e.g., “The boy read the book,” which does not require complement coercion, and “The girl slept until dawn,” which does not require iteration) in Broca's and Wernicke's aphasics. Patients listened to a sentence and saw two pictures: they had to choose the one that matched the sentence in content. In all cases, Broca's aphasics performed above chance, but Wernicke's aphasics were at chance for both complement and aspectual coercion. Consistent with this finding, and with lesions in the posterior superior and middle temporal gyri (pMSTG, and neighbouring brain areas) in Wernicke's aphasics, Brennan and Pylkkänen (2008) and Pylkkänen and McElree (2007) using MEG observed recruitment of temporal and ventromedial prefrontal cortices around 450 ms from the onset of critical words in complement and aspectual coercion. Other studies, however, suggest different cortical mechanisms for complement and aspectual coercion, with complement coercion modulating at least the N400 amplitude (Baggio et al., 2010; Kuperberg et al., 2010), and aspectual coercion resulting in larger sustained negativities (Paczynski et al., 2014) similar to the ERP effects found in other cases of aspectual processing (Baggio et al., 2008).

Previous experimental research has focused largely on a single type of aspectual coercion (i.e., coercing a punctual into an iterative event). Still, the results show that coercion implies *on-line* processing costs. Three issues have been relatively underexplored. First, what would semantic theory predict for *off-line* responses patterns, e.g., in a two-alternative forced-choice task where participants are given a sentence of the types (1)–(9), and are asked to decide whether it describes a “possible event” or not? Second, how do these response patterns compare *across* aspectual coercion types: specifically, are sentences involving subtractive and cross coercion more difficult and less acceptable, as suggested by the proposals of Koontz-Garboden (2007) and Michaelis (2003, 2004)? Third, how do ALS patients understand sentences involving aspectual coercion? Would the motor neuron disease have a measurable effect on event structure representations, on coercion operations, or on constraints on their application? Three hypotheses were tested in our study using a simple binary response task that patients with ALS and motor or speech impairment would still be able to perform: provide “yes” or “no” answers to the question whether a sentence, of the types (1)–(9), describes a “possible event.” Healthy controls are expected to perform at or near ceiling level (in any case above chance) in constructions that do not involve coercion, while performance with aspectual coercions should be closer to chance, reflecting complexity in the application of constraints on interpretation. For ALS patients, the hypotheses are:

- If representations of event structures are damaged, interpretations would be either semantically empty or disorganized; therefore, responses should be at chance level for all sentence types, including sentences that do not involve aspectual coercion;
- If aspectual coercion operations are damaged, the mismatch between the event structure associated with the VP and the semantics of the temporal PP cannot be resolved; all sentences that require coercion should be understood as anomalous, and should therefore lead to lower-than-chance proportions of affirmative response; responses should still be close to ceiling in sentences that do not involve coercion;
- if constraints on coercion are damaged, event structures are transformed according to the requirements of temporal modifiers, regardless of whether the result is consistent with lexical and event knowledge; thus, in sentences requiring coercion assent rates should be higher than in controls.

## Methods

### Participants

Thirteen patients with a diagnosis of ALS participated in the study (5 women; age *M* = 65.4 years, *SD* = 10.1; education *M* = 9.15 years, *SD* = 4.74). Eleven were recruited at the Neurology Unit of the University Hospital in Udine (Azienda Ospedaliero Universitaria Santa Maria della Misericordia), and the remaining two were recruited at the Neurology Unit at the Cattinara Hospital in Trieste (Azienda Ospedaliero Universitaria Ospedali Riuniti). Only patients whom a neurologist deemed able to perform the task were included in the study. The only additional inclusion criterion was absence of a diagnosis of dementia. A neurologist assessed the degree of functional ability of patients by means of the ALSFRS-R (ALS Functional Rating Scale, Revised; Cedarbaum et al., 1999; Table 2). Four patients had bulbar-onset ALS, nine had limb-onset ALS. Seven patients showed (mild or severe) signs of dysarthria. All patients were being treated with riluzole. Two patients (Cases 4–5, Table 2), for whom MR images were available (see Section Imaging), were assessed using standard neuropsychological batteries: the Mini-Mental State Examination (MMSE; Folstein et al., 1975); the Aachener Aphasia Test (AAT, Italian version; Luzzatti et al., 1996); the grammaticality judgment and the grammatical comprehension tests of BADA (Batteria per l'Analisi dei Deficit Afasici; Miceli et al., 1994); ideational and ideomotor apraxia tests (De Renzi et al., 1980; De Renzi and Lucchelli, 1988); Poppelreuter-Ghent's Overlapping Figures test (Della Sala et al., 1995; Table 6). Patients were native speakers of Italian. As a control group we recruited 13 Italian native speakers (7 women; age *M* = 64.6 years, *SD* = 5.84; education *M* = 9.92 years, *SD* = 3.84), with no history of neurological disorders or trauma, residents of the same region in North-Eastern Italy as ALS patients. Controls and ALS patients were matched for age (Wilcoxon rank sum test, continuity correction: *W* = 84, *p* = 1) and education (*W* = 69.5, *p* = 0.4472).

**Table 2:** **Demographic and clinical information about the ALS patient group**.

**Case**	**Gender**	**Age**	**Education (years)**	**Test from ALS onset (months)**	**ALSFRS-R**	**ALS onset site**
1	M	62	8	19	30	Bulbar
2	F	79	5	8	42	Bulbar
3	M	64	10	6	38	Bulbar
4	F	80	7	27	29	Limb
5	M	78	5	5	20	Limb
6	M	63	22	16	42	Limb
7	F	56	12	15	44	Limb
8	M	74	5	21	33	Limb
9	F	62	5	14	30	Limb
10	M	63	8	36	30	Limb
11	F	58	11	14	25	Limb
12	M	66	8	36	43	Limb
13	M	45	11	12	39	Bulbar

*ALSFRS-R is the ALS Functional Rating Scale (Cedarbaum et al., 1999). The scores range from 0 to 48 (best)*.

All participants were informed about the purposes and duration of the experiment. All patients signed an informed consent form that stated that the study had no clinical or therapeutic goals or consequences, and that they had the right to quit the study at any time. The study was approved by the Ethics Committee of the International School for Advanced Studies (SISSA, Trieste). Patients were asked whether they agreed to being recorded during the task, after being reassured that the audio files would be anonymized and used for research purposes only: 6 patients agreed, 7 declined. Upon request of some patients, a partner or spouse was present in the testing room. In such cases, the couples were explicitly requested to avoid communicating during testing. No participant withdrew, and all patients and controls completed the test.

### Materials

The stimuli were constructed from a set of 16 nouns in Italian, drawn from 4 semantic categories: media (“television,” “radio,” “computer,” and “newspaper”), transport (“car,” “bus,” “train,” and “bike”), food or drinks (“cake,” “coffee,” “apple,” and “pasta”), and tools (“keys,” “knife,” “pencil,” and “screwdriver”). For each noun a set of 3 verbs was generated, one from each aspectual class: e.g., for “television” the verbs were “to watch” (activity), “to switch on” (achievement), and “to repair” (accomplishment). Combining each noun with its 3 associated verbs resulted in 48 verb phrases (VPs) and each VP was combined with 3 modifier phrases, constructed using 3 prepositions: “for [X time],” “in [X time],” and “at [Y time].” The extent or the temporal location of the time interval or point denoted by each temporal phrase was tuned to a plausible duration or location in time of the event described by the VP given its aspectual class as is normally (outside aspectual coercion contexts) modified by the phrase: e.g., using “to watch the television” as a base, the phrase “for half an hour” was used; and “to switch on the television” + “at noon,” and “to repair the television” + “in half an hour.” As in previous experimental studies, the [X time] phrase was the same with “in” and “for” modifiers (e.g., “half an hour”). Combining the 48 VPs with each of 3 modifier phrases resulted in a set of 144 sentences (the final stimulus list), divided into 9 sentence types (Table 1): 3 non-coercing classes (ACT↝ACT, ACH↝ACH, ACC↝ACC) and 6 coercions: 2 additive (ACT↝ACC, ACH↝ACC), 2 subtractive (ACC↝ACT, ACC↝ACH), 2 cross (ACT↝ACH, ACH↝ACT).

The materials were normed using the CORIS/CODIS corpus of written Italian (Rossini Favretti et al., 2002). The corpus was queried for the raw frequency of verbs (e.g., “guardare”) and of VPs (e.g., “guardare la televisione”), allowing for up to 3 intervening words between the V and the NP (to include negation and various modifiers: e.g., “[…] guardare mai la televisione […]” and “[…] guardare un po' di televisione […]” were counted as positive instances of the VP). There was no difference in raw verb frequency across aspectual classes (Wilcoxon rank sum tests: ACT/ACH, *W* = 160, *p* = 0.2; ACT/ACC, *W* = 163, *p* = 0.2; ACH/ACC, *W* = 140, *p* = 0.67). There was no difference in the raw frequency of VPs across aspectual classes (Wilcoxon rank sum tests, continuity correction: ACT/ACH, *W* = 134.5, *p* = 0.82; ACT/ACC, *W* = 137.5, *p* = 0.73; ACH/ACC, *W* = 129.5, *p* = 0.97).

Thirteen booklets were printed with a different order of presentation of the 144 sentences. The sentences were presented in a list (~30 per page, 5 pages per booklet) with a bounded space on the right-hand side for recording participant responses. The cover page of the booklet stated the instructions: “This booklet contains a list of sentences. Please read each sentence carefully. Your task is to decide whether each sentence describes a possible event. Here you should decide whether each event is possible in general, not whether it is something you are able to do, or something you do on a regular basis. Please note that no sentence is repeated twice, but some sentences differ by one or a few words only. One must pay attention to all words in a sentence. There are no right or wrong answers.” The latter qualification also explains why we use “affirmative” and “negative” as response labels through this paper, instead of “correct” and “incorrect.”

### Procedure

Patients were tested in a quiet room in the premises of the neurology units of the University Hospital in Udine or the Cattinara Hospital in Trieste (see Section Participants). The patient and experimenter sat in front of each other at a regular desk. The experimenter read the instructions aloud from the cover page of the booklet, and invited the patient to read and sign the informed consent sheet (Section Participants). The patient was asked to read each sentence from the booklet, and respond with a “yes” or “no” to the specific question posed on the booklet's cover page. Most patients with no or weak dysarthria spontaneously read aloud the sentences. Two ALS patients were unable to do so, and were aided by the experimenter, who read aloud each sentence. However, these patients were able to produce “yes” or “no” answers, and only occasionally answered with head movements. The experimenter monitored the patient's progression in the task on a laptop computer, where the patient's responses were recorded (also as an audio file, if the patient had to agreed to being taped). Reading was either self-paced by the patient, or adjusted by the experimenter to possible signs of fatigue in the 2 dysarthric patients who could not (consistently) read sentences aloud. The experimenter intervened to ask why the patient had given a certain response in a subset of all trials (~10 per ALS patient) where responses followed signs of hesitation, or were negative in no-coercion cases or in predictably difficult cases of coercion (i.e., subtractive and cross coercion). In all cases, the query by the experimenter was limited to a *why*-question. The patient's explanation was never followed up by further questions or feedback by the experimenter.

### Imaging

MRI scans of 2 ALS patients (Case 4 and Case 5; see Table 2) were acquired at the Operative Clinical Unit of Radiology (MRI section) at Cattinara Hospital in Trieste. MR imaging was performed using a 1.5T Philips Achieva system with a 16-channel coil. T2 and Fluid-Attenuated Inversion Recovery (FLAIR) pulse sequences were used, covering the whole brain and encephalic trunk. The T2 sequence had TR = 2500 ms and TE = 80 ms. The FLAIR sequence had TR = 8000 ms, TE = 100 ms, and 2500 ms inversion time. Using T2 and FLAIR sequences, 22 axial slices (5-mm volume thickness, 5.5-mm between-slice spacing) were acquired. MR images were obtained shortly before testing took place.

### Data analysis

Response data were analyzed using the same procedure for ALS patients and controls. For each of the 9 sentence types (Table 1), we summed the number of affirmative responses given by each participant; the sum ranges from 0 to 16: the total of trials for each type. Means were computed for each condition, defined in three alternative ways: (a) according to *aspectual coercion classes*: null, additive (ACT↝ACC and ACH↝ACC), subtractive (ACC↝ACT, ACC↝ACH) and cross coercion (ACT↝ACH, ACH↝ACT); (b) according to *input aspectual classes*: activity (ACT↝X coercions), accomplishment (ACC↝X), achievement (ACH↝X); finally, (c) according to *output aspectual classes*: activity (X↝ACT), accomplishment (X↝ACC), achievement (X↝ACH). Means were computed in each experimental condition, and in each group (ALS patients and controls). We analyzed the data using three ANOVA models (see Table 3) for (a), (b), or (c). All three models comprised one between-subjects Group factor (2 levels: ALS or control), one within-subjects Coercion factor (4 levels in model (a): null, additive, subtractive and cross; 3 levels in (b)–(c): activity, achievement, and accomplishment), and Affirmative Responses as a dependent variable. To discount the effects of generic response biases on judgments (e.g., due to the testing environment and other factors), we analyzed the data considering the null coercion condition as a baseline: the number of affirmative responses to non-coercing sentences (*n*) for each participant (ALS patient or control) was subtracted from the number of affirmative responses in each of the coercing conditions (*c*). In each condition the mean of raw binary responses over trials was replaced by a corrected value *c*–*n*. The data series were symmetric in all cases (*m*-out-of-*n* bootstrap symmetry tests, *p* > 0.05; Miao et al., 2006). Some data series had non-normal distributions (Shapiro-Wilk tests, *p* < 0.05), hence we compared patients and controls using non-parametric Wilcoxon rank sum tests with continuity correction (Tables 4, 5). Permutation-based *t*-tests (for unpaired samples) were also applied in the same comparisons: in cases, such as the present study, where sample size is small, the assumptions of standard parametric statistics cannot be subjected to valid tests, hence non-parametric alternatives (e.g., permutation tests) should be preferred (Siegel, 1956, 1957).

**Table 3:** **Results of ANOVA statistics**.

	**Group**	**Coercion**	**Group × Coercion**
(a) Coercion types	*F*_(1, 24)_ = 10.37	*F*_(2, 48)_ = 13.832	*F*_(2, 48)_ = 0.772
	*p* = 0.0037	*p* < 0.0001	*p* = 0.514
(b) Input aspectual class	*F*_(1, 24)_ = 12.58	*F*_(2, 48)_ = 27.632	*F*_(2, 48)_ = 0.229
	*p* = 0.0016	*p* < 0.0001	*p* = 0.796
(c) Output aspectual class	*F*_(1, 24)_ = 12.58	*F*_(2, 48)_ = 16.757	*F*_(2, 48)_ = 0.123
	*p* = 0.0016	*p* < 0.0001	*p* = 0.884

*Three models were used, with different constructions of the within-subjects Coercion factor: (a) across types of coercions (4 levels: null, additive, subtractive, cross), (b) across the aspectual class of the VPs to be coerced, or input classes (3 levels: activity, accomplishment, achievement), and (c) across the aspectual classes resulting from coercion, or output classes (3 levels: activity, accomplishment, achievement)*.

**Table 4:** **Results of Wilcoxon rank sum tests with continuity correction comparing the ALS and control groups on affirmative answers in particular experimental conditions**.

	**ALS**	**Control**	***d***	**W**	***p***	
No coercion	*M* = 14.10, *SD* = 2.06	*M* = 11.62, *SD* = 3.81	0.812	128.5	0.0253	
Additive coercion	*M* = 11.54, *SD* = 2.93	*M* = 7.81, *SD* = 3.47	1.161	128.5	0.0254	a
Subtractive coercion	*M* = 13.46, *SD* = 2.38	*M* = 9.77, *SD* = 3.80	1.165	139	0.0055	b
Cross coercion	*M* = 12.62, *SD* = 1.56	*M* = 9.77, *SD* = 2.93	1.214	147.5	0.0013	
Activity↝X	*M* = 13.77, *SD* = 2.29	*M* = 10.27, *SD* = 2.70	1.399	140.5	0.0043	
Accomplishment↝X	*M* = 13.46, *SD* = 2.38	*M* = 9.77, *SD* = 3.80	1.165	139	0.0055	b
Achievement↝X	*M* = 10.38, *SD* = 2.03	*M* = 7.31, *SD* = 3.29	1.126	129	0.0227	
X↝Activity	*M* = 11.73, *SD* = 2.47	*M* = 8.35, *SD* = 3.29	1.162	137	0.0075	
X↝Accomplishment	*M* = 11.54, *SD* = 2.93	*M* = 7.81, *SD* = 3.47	1.161	128.5	0.0254	a
X↝Achievement	*M* = 14.35, *SD* = 1.72	*M* = 11.19, *SD* = 3.67	1.1	131	0.0168	

*Means (M), standard deviations (SD) and effect sizes (Cohen's d) are also shown. Additive coercion and X ↝ Accomplishment coercion (a) coincide, and the same applies to subtractive coercion and Accomplishment ↝ X coercion (b), hence identical statistical values in the table. Values are rounded to the second decimal figure*.

**Table 5:** **Results of Wilcoxon rank sum tests with continuity correction comparing the ALS and control groups on affirmative answers (minus the no-coercion baseline)**.

	**ALS**	**Control**	***d***	**W**	***p***	
Additive coercion	*M* = −2.56, *SD* = 2.15	*M* = −3.81, *SD* = 4.28	0.367	98	0.505	a
Subtractive coercion	*M* = −0.64, *SD* = 1.01	*M* = −1.85, SD = 1.39	0.992	128	0.027	b
Cross coercion	*M* = −1.49, *SD* = 1.31	*M* = −1.85, *SD* = 2.01	0.212	94.5	0.625	
Activity↝X	*M* = −0.33, *SD* = 1.64	*M* = −1.35, *SD* = 3.02	0.416	102	0.382	
Accomplishment↝X	*M* = −0.64, *SD* = 1.01	*M* = −1.85, *SD* = 1.39	0.992	128	0.027	b
Achievement↝X	*M* = −3.72, *SD* = 1.42	*M* = −4.31, *SD* = 3.26	0.234	96	0.572	
X↝Activity	*M* = −2.37, *SD* = 1.31	*M* = −3.27, *SD* = 2.4	0.465	103	0.355	
X↝Accomplishment	*M* = −2.56, *SD* = 2.15	*M* = −3.81, *SD* = 4.28	0.367	98	0.505	a
X↝Achievement	*M* = 0.24, *SD* = 1.43	*M* = −0.42, *SD* = 1.24	0.498	111.5	0.173	

*Means (M), standard deviations (SD) and effect sizes (Cohen's d) are also shown. Additive coercion and X ↝ Accomplishment coercion (a) coincide, and the same applies to subtractive coercion and Accomplishment ↝ X coercion (b), hence identical statistical values in the table. Values are rounded to the second decimal figure*.

**Table 6:** **Neuropsychological assessment of Case 4 and Case 5**.

		**Cutoff-max**	**Case 4**	**Case 5**
MMSE		27–30	28,7	27,7
AAT	Spontaneous speech	5-4-5-5-5	5-3^*^-5-5-5-5	5-3^*^-5-5-5-5
	Token test	43–50	50	46
	Repetition	137–150	147	138
	Written language	61–90	88	88
	Naming	108–120	120	119
	Comprehension	90–120	118	104
BADA	Grammaticality judgments	errors/48	1	3
	Grammatical comprehension	errors/60	2	4
	Ideational apraxia	14–14	13^*^	14
	Ideomotor apraxia	53–72	47^*^	68
	Overlapping figures	*x*/71	70	69

*The middle column shows the maximum score in each subtest (or the maximum number of errors possible) with the cut-off score for participants tested in the hospital, where applicable. MMSE is the Mini-Mental State Examination (Folstein et al., 1975), AAT is the Aachener Aphasia Test (Luzzatti et al., 1996), BADA is the Battery for the Analysis of Aphasic Deficits (Miceli et al., 1994). These were followed by tests for ideational and ideomotor apraxia (De Renzi et al., 1980; De Renzi and Lucchelli, 1988), and by Poppelreuter-Ghent's Overlapping Figures Test (Della Sala et al., 1995). Performances below the cut-off point are marked in bold and with an asterisk. In the AAT score reports, the second score (marked with an asterisk in both patients) is for articulated speech*.

## Results

Below we first report the results of the main experiment for ALS patients and controls (Section Response Patterns in Patients and Controls). We observed significant differences in assent rates (number of affirmative answers) in response to coercing sentences between groups: ALS patients showed higher assent rates, particularly when coercion required the deletion of elements from an event structure (subtractive coercion). Further, we describe two case studies drawn from our patient sample, combining data from the main task, in-depth neuropsychological testing and MRI scans (Section Case studies). A sample of the dialogues we engaged in with patients, providing qualitative information on their understanding of coercion, is presented as an Appendix in Supplementary Material.

### Response patterns in patients and controls

The number of affirmative responses was overall higher in ALS patients than in controls (Tables 1, 4, 5): there was an effect of Group in all ANOVA models (Table 3). There were differences in the proportion of affirmative responses across conditions, evidenced by a significant effect of Coercion in the ANOVA (Table 3). However, the response pattern was qualitatively similar in patients and controls: we found no interactions between Group and Coercion (Table 3). Therefore, across conditions, the responses of ALS patients and controls to different sentence types seem comparable, with the main difference being that patients show a higher number of affirmative responses overall.

**Figure 1:**
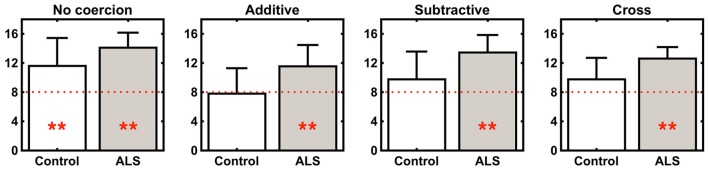
**Plots showing the means of affirmative responses (bar height) and standard deviations (whisker length) for controls and ALS patients in the different experimental conditions**. Red lines show chance levels, and asterisks indicate statistically significant effects (at *p* < 0.01) in Wilcoxon one-sample tests against chance (8 affirmative responses in 16 trials; see Results for details).

A closer look at the data, however, comparing patients and controls in each experimental condition separately, reveals subtle but robust differences in response patterns. In no-coercion items [examples (1), (6), (8) in Table 1], the proportion of affirmative responses is above chance in ALS patients and in controls (one-sample Wilcoxon signed rank tests, *p* < 0.01). Responses drop down at chance in controls in additive (*V* = 36.5, *p* = 0.552), subtractive (*V* = 66, *p* = 0.161) and cross coercions (*V* = 75, *p* = 0.042) (Tables 1, 4). Strikingly, in ALS patients, responses remain well *above chance* in additive (*V* = 85.5, *p* = 0.006), subtractive (*V* = 90, *p* = 0.002) and cross coercions (*V* = 91, *p* = 0.002) (Figure 1; Tables 1, 4). Since ALS patients gave significantly more affirmative response than controls also in no-coercion items (*W* = 128.5, *p* = 0.025), it is necessary to compare performance across groups discounting this difference. An analysis of baseline-corrected data (Methods, Data Analysis) confirmed that ALS patients accept more instances of subtractive coercion (i.e., ACC↝X) than controls, relative to each group's baseline level (Table 5). We performed permutation *t*-tests on baseline-corrected data: these show that the only difference between groups surviving this conservative baseline correction concerns subtractive coercion (*t* = 2.53, *p* = 0.018; all other *p* > 0.2). We did not find any correlations between ALS patient's response frequencies and age, education, time of testing from ALS onset, and ALSFRS-R scores.

### Case studies

We further assessed general cognitive capacities, and the presence of signs of aphasia and apraxia, in 2 ALS patients for whom MR scans could be acquired. We used a series of subtests from standard neuropsychological batteries: the Mini-Mental State Examination (MMSE; Folstein et al., 1975), in the form of a 30-point questionnaire assessing cognitive functions; the Aachener Aphasia Test (AAT; Luzzatti et al., 1996), including a Spontaneous Speech Test (a brief semi-structured interview), the Token Test (patients give gestural responses to verbal commands, e.g., “touch the red square”; the length and the syntactic complexity of commands increases in 5 subsequent parts), a Repetition Test (of sounds, single-syllable and multisyllable words, morphologically complex words, and sentences), a Written Language Test (of reading aloud words and phrases, composing words and phrases, writing dictated words and phrases), a Naming Test (of objects, colors, pictured compound nouns and sentences), and a Comprehension Test (of spoken and written words and sentences; the patient points to the correct target choosing among 4 pictures, 2 of which are distractors, 1 is unrelated). Grammar was further assessed using the auditory grammaticality judgment and the grammatical comprehension tests of BADA (Miceli et al., 1994). ALS patients' ability to demonstrate the use of everyday objects (e.g., a hammer, a toothbrush, a comb etc.) and to imitate finger and hand movements (e.g., cutting with scissors, the “OK” sign etc.) was assessed by means of tests for ideational and ideomotor apraxia (De Renzi et al., 1980; De Renzi and Lucchelli, 1988). Poppelreuter-Ghent's Overlapping Figures Test (Della Sala et al., 1995) was used to assess patients' ability to recognize visual objects in figures containing two or more partly overlapping object drawings.

**Figure 2:**
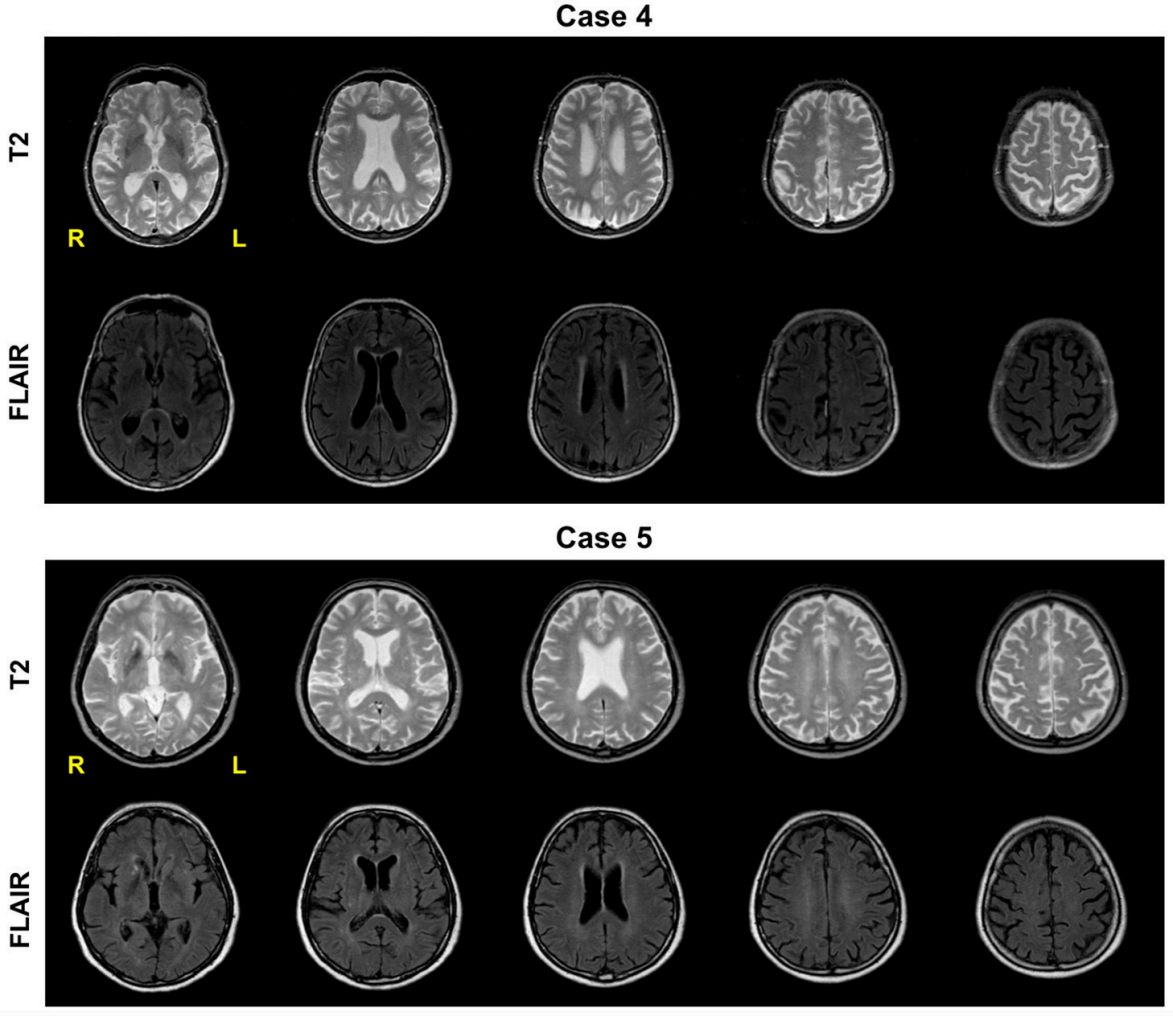
**Axial T2-weighted (top) and FLAIR (bottom) MR images for Case 4 and Case 5**.

Case 4 is a 80-year-old woman with 7 years of education, and a score of 29 in the ALSFRS-R. She was tested 27 months after ALS (limb) onset. The patient performs well (i.e., within the normal range) in all AAT subtests, but she presents the classical signs of dysarthria: her spontaneous speech is slow and labored. She does not have difficulty in finding words and in organizing sentences syntactically. The patient has spared morphosyntactic competence, and commits only few mistakes in the auditory grammaticality judgment and comprehension BADA tests. Her performance in the Overlapping Figures Test is nearly flawless. There is some evidence of ideomotor apraxia, but few clear signs of ideational apraxia: the patient can demonstrate the use of objects in most cases, but she has some difficulty imitating hand and finger movements. As remarked by Papeo et al. (2015b), the effects of ALS on the motor system's periphery might explain poor performance here. Case 4 is representative of the aged ALS population in that she presents no symptoms of dementia and cognitive impairment, and no evidence of aphasia, whereas she displays the typical ALS manifestations of dysarthria and possibly apraxia. Her MR scans are in line with this neuropsychological and clinical picture. Her brain shows diffuse cortical atrophy, with an enlargement of subarachnoid spaces, basal cisternae and ventricular cavities, with periventricular white matter disease (a possible result of white matter ischemia, common in aged individuals), and evidence of gliosis in subcortical white matter (Figure 2, top panel). There is a focus of T2 hyperintensity in right parietal cortex, which might explain the patient's poor score in the action imitation test (Sirigu et al., 1996; Iacoboni et al., 1999). The patient's performance in the main aspectual coercion task was within one standard deviation from the group's mean. Patient-specific means were used as μ values in non-parametric one-sample Wilcoxon tests in which the data series is given by the ALS patient group, minus Case 4: all these tests yielded non-significant results (*p* > 0.1), suggesting case-specific performance values are close to the group's mean.

Case 5 is a 78-year-old man with 5 years of education, and a score of 20 in the ALSFRS-R. He was tested 5 months after ALS (limb) onset. As Case 4, the patient exhibits the typical signs of dysarthria (slow, effortful speech), but semantic and syntactic aspects of spontaneous speech are largely intact. The patient commits 4 mistakes in the more complex trials of the token test. His performance is however above the cut-off for non-aphasic patients tested in the hospital in all remaining AAT tests, that is, repetition, written language, naming and comprehension (Miller et al., 2000). The patient scores well (and within the normal range) in the object manipulation and movement imitation tasks, as well as in the overlapping figures test. Case 5 is another example of an aged non-demented non-aphasic ALS patient with dysarthria and scarce physical ability (as evidenced by his ALSFRS-R score). As with Case 4, his MR scans present the typical pattern of aged ALS patients: expanded ventricles, ischemic periventricular white matter, wider subarachnoid spaces and basal cisternae, subcortical white matter gliosis, and generalized cortical atrophy. Here too, the patient's performance in the main task is within one standard deviation from the group's mean. Patient-specific means were used as μ values in one-sample Wilcoxon tests where the sample data series is from the ALS group, minus Case 5: all tests returned non-significant effects (*p* > 0.1).

## Discussion

We investigated the comprehension of sentences containing combinations of different types of verb phrases (VPs) and temporal modifiers in ALS patients and controls: the VPs could be from either of three different aspectual classes (or *Aktionsarten*: activities, e.g., “watch the television”; accomplishments, e.g., “repair the television”; achievements, e.g., “switch on the television”), followed by either of three types of temporal prepositional phrases (“for half an hour,” which combines preferentially with activities; “in half an hour,” which is used with accomplishments; “at noon,” used with achievements). However, each of the VP types may, in principle, be combined with any of the temporal PPs, but this form of “free combination” requires that the meaning of the relevant VP (i.e., its associated event structure) is *modified* so as to suit the semantics of the temporal PP. In the formal semantic literature, the modifying operation is referred to as aspectual coercion. Although (considering 3 aspectual classes and 3 temporal modifiers) there are 6 possible transformations, we classified aspectual coercions into 3 types: i.e., additive coercions (where elements are added to event structures, e.g., a process or a goal), subtractive coercions (in which event structure elements are removed), and cross coercions (involving both deletion and addition of elements). Healthy controls rejected sentences that involved aspectual coercion more often than sentences where VPs were used with their preferred modifiers. The same pattern was observed in ALS patients. However, patients showed a larger proportion of accepted instances of aspectual coercion across conditions than controls, in particular when the deletion of event structure elements was involved (subtractive coercion). We put forward the hypothesis, based on Cases 4 and 5, that this effect can occur in ALS patients in the absence of generalized cognitive decline, dementia and aphasia, regardless of whether or not the patient is apraxic. In what follows, we discuss three sets of issues: (1) whether ALS, as a disease of the human motor system, is an appropriate model for testing embodied semantics and related hypotheses; (2) whether ALS could serve as a model for studying discourse processes, such as the role of knowledge-based inferences and of constraints on the application of (aspectual) coercion and other semantic operations; (3) whether standard neuropsychological batteries, especially those designed to identify signs of aphasia, may be integrated with tests for specific operations, drawing on insights from formal syntax and semantics.

### Verb phrase semantics and the motor system

As explained in the Introduction, the interpretation of sentences containing a VP and a temporal modifier, in particular when the VP is *not* accompanied by its preferred preposition (e.g., activities by “for” PPs) requires the integrity of three structures, postulated by formal semantic theory: (i) a *representation* of the meaning of the VP specifying the default (i.e., absent coercion) causal and temporal profiles of the relevant events, or “event structure”; (ii) a finite set of *operations* that transform the default event structure into a new structure by adding and/or removing elements; these operations (e.g., aspectual coercion) are triggered by particular temporal modifiers; (iii) a set of *constraints* on the applicability of operations on event structures, drawing on lexical knowledge (i.e., the meaning of verbs and VPs cannot be stretched much beyond what is specified in lexical structures) and event knowledge (the transformed event representations should be consistent with what one knows about the typical duration of certain events, their causes and effects). On the assumption that each of these components can be damaged independently, and that no other “central process” may affect the interpretation of temporal VP+PP sentences, three predictions ensue. First, damaging representations of event structures should lead to *chance-level* acceptability judgments in a task such as ours: if the meaning of the VP cannot be accessed and retrieved, there is no input to the transformation, and no material for a discourse model to be constructed; hence, responses should be random. Second, damaging coercion operations should yield a majority of *negative* acceptability judgments: if the input event structure is left untouched, combinations with non-preferred PPs will always lead to inconsistent discourse models; thus, patients should reject all or most putative cases of coercion. Third, damaging constraints on the application of coercion should result in a majority of *positive* acceptability judgments: if the default lexical semantics of the VP can be modified “freely,” and if the outcome of the transformation of default event structures need not cohere with stored knowledge, combinations with non-preferred PPs will always give consistent discourse models; patients should therefore accept most cases of coercion. In what follows, we will address each of these predictions in turn.

Previous research has used ALS to test a number of hypotheses on the functional links between the semantics of action and action verbs, and bodily action and movement representations in the motor system (Bak and Chandran, 2012). Some of these studies described deficits in ALS in the production and comprehension of verbs as compared to nouns, in naming pictures of actions relative to pictures of objects, in matching a verb with a corresponding action compared to matching a noun with its referent, and in matching picture pairs involving actions compared to pictures of objects (Bak and Hodges, 1997, 2004; Bak et al., 2001). These results seemed to suggest that action semantics, whether it is accessed through language or otherwise, is impaired in ALS. Because ALS results in the progressive atrophy of both peripheral and central motor systems, these findings were taken to imply a link between the motor system and action semantics, whereby the latter relies functionally on the former, as in models of embodied semantics (Bak and Chandran, 2012). This conclusion has been challenged by Papeo et al. (2015b), who found that ALS patients show a selective deficit of action organization, whereas processing of action verbs is largely intact. We did not test ALS patients for production or comprehension of single verbs and VPs, but the interpretation of stimulus sentences in our experiment does presuppose spared VP representations. As we noted above, if event structures associated to VPs (stored in the lexicon or computed “on the fly”) were deteriorated or absent in ALS, we should observe chance-level performance in our semantic acceptability judgment task. As we did not see such pattern, and we have moreover evidence from our dialogues with patients (see Appendix in Supplementary Material) that they fully understand the meaning of VPs (indeed of whole sentences), we conclude that VP semantics is largely spared in ALS. Our data side with Papeo et al. (2015b) in providing indirect evidence for the relative independence of V or VP semantics from representations and processes residing within the motor system (for recent work challenging the embodied hypothesis, see Pavan and Baggio, 2013; Pavan et al., 2013; Papeo et al., 2015a; Bottini et al., 2016; Ghio et al., 2016; Areshenkoff et al., 2017).

### Inference and constraints on interpretation

Not only do these ALS patients present a pattern of behavior consistent with intact VP representations: they are also able to turn a default event structure, associated with an input VP, into a modified structure satisfying the semantic requirements set by temporal PPs. If ALS patients were unable to aspectually coerce VPs, we should see a high number of rejections in sentences requiring coercion (if they are to result in coherent discourse models): that was not the case here. If anything, there was a marked response tendency in the opposite direction. Our dialogues with patients provide further evidence that they are able to deploy aspectual coercion operations (see Appendix in Supplementary Material). The problem is rather the opposite: ALS patients accept coercing constructions significantly more often than controls. Why might this be the case?

Having excluded a disorder of lexical event knowledge in ALS patients (which should have produced chance-level performance) and an impairment of coercion operations (which would have yield floor-level responses), might it be that semantic representations of the temporal prepositions “in,” “for” and “at” are damaged in ALS? It seems we can exclude that, because ALS patients perform above chance in all output class conditions (i.e., X↝ACT, *for*-phrases; X↝ACC, *in*-phrases; and X↝ACH, *at*-phrases), and they are able to correct the form of coercing sentences by proposing specific substitutions (e.g., replacing “in” with “for” if the VP denotes an activity; Appendix in Supplementary Material). Another possibility is that constraints on coercion are compromised in ALS. Although patients may be able to draw inferences relating event structures to event knowledge (see Appendix in Supplementary Material), they may not always use their conclusions to determine whether aspectual coercion can be applied in each case. This failure would result in an *overapplication of coercion*, beyond what controls normally do. We favor the latter explanation here, albeit tentatively, for the following two reasons. First, performance differences between patients and controls are counter-intuitive as they are *not* consistent with what would be predicted by known effects of neurological disorders: impaired lexico-semantic knowledge, or impaired use of structural (syntactic and semantic) operations. We ruled out both options, based on acceptability data (neither at chance nor at floor levels) and on data from our dialogues with ALS patients (Appendix in Supplementary Material). A different account should be invoked to explain the observed effects. Second, the only alternative view contemplated by theories of coercion (Pustejovsky and Bouillon, 1995; Pulman, 1997; Michaelis, 2003, 2004; Koontz-Garboden, 2007; Dölling, 2014) is a *deficit of constraints on coercion*. There is no other way to explain, within a formal semantic theory, performance near ceiling across aspectual coercion types. This hypothesis should be tested in further work. If ALS does not affect compositional processes (lexical structures and semantic combinatorics), but mainly the selection of compositionally-generated alternatives, we might find similar effects of relaxed or absent constraints on interpretation in discourse and pragmatic processes, consistent with earlier work (Staios et al., 2013; Ash et al., 2014; Bambini et al., 2016).

However, there are more factors that affect processing than semantic theories can envisage. Two such extra-theoretic alternative explanations are particularly relevant here. One is based on the observation that ALS patients perform worse than controls in executive function tasks (Phukan et al., 2007, 2011; Consonni et al., 2013; Goldstein and Abrahams, 2013). Some of these findings are counterintuitive. For example, in a computerized Tower of Hanoi task, patients with ALS and pseudobulbar palsy, relative to controls and ALS patients without pseudobulbar palsy, exhibit *shorter* planning times and more errors in more complex trials (Abrahams et al., 1997). This pattern shows that some ALS patients “rush ahead” toward flawed solutions. A similar tendency may explain the bias toward affirmative responses in our data. Only few ALS patients in our sample showed the signs of pseudobulbar palsy (i.e., dysarthria or dysphagia, in particular), and we have no evidence of executive dysfunction in the patients tested here. Further work should try to correlate executive (dys)function in ALS with performance in advanced semantic tasks, e.g., involving coercion. The current literature indeed attempts to understand if the well-known dysexecutive syndrome in ALS underlies cognitive deficits, including language. The present study cannot contribute to this debate.

Another alternative explanation of our results is that ALS patients (or patients, or participants, in general) tested in the hospital show a bias toward affirmative responses in forced-choice tasks such as ours perhaps as a way of signaling cooperativeness with researchers. If supported by further evidence, this would be a novel result in itself. To our knowledge such “framing effects” in the context of neuropsychological testing have not been described to date. This issue should be addressed using different formulations of the same task (alternating affirmative and negative questions, or using rating scales instead of binary questions). However, what is known and more plausibly assumed is that the hospital setting produces more *random errors*, and not more positive responses: that is typically taken into account when defining cut-off points in neuropsychological tests (Miller et al., 2000). On the other hand, regardless of whether these accounts explain the generic bias toward positive responses in the present ALS sample, they do not explain why subtractive coercion shows the largest between-group difference also with baseline-corrected data. Also, if there was a *generic* bias toward producing affirmative responses (one *not* specific to semantic processing, whether this bias is produced by the hospital setting, by executive dysfunction or other factors), the same bias should also apply to similar tasks, e.g., to the BADA grammaticality judgment subtest. But this was not the case: if a generic bias applied, ALS patients would produce more errors than those reported in Tables 1, 4, 5.

One may capture these rather subtler effects within a semantic theory. As noted in the Introduction, Koontz-Garboden (2007) proposed that, among other constraints on coercion, *monotonicity* allows elements to be added, but not to be deleted from an event structure. The prediction here is that additive coercions are more acceptable than both subtractive and cross coercions. Yet, we found the opposite pattern in both ALS patients and controls: subtractive and cross coercion are accepted more often than additive coercion. Michaelis's (2003) constraint of *Aktionsart preservation* predicts cross coercion to be the most complex case, involving two steps: first deletion, then addition, or vice versa. In our study, we found subtractive and cross coercion to follow a similar pattern. ALS may have different (although non-selective) effects on constraints on aspectual coercion, such that the performance of ALS patients appears more monotonic (i.e., less sensitive to information that would block coercion) than in controls, in cases that require the deletion of elements from event structures. This is a hypothesis that requires further testing: it predicts that ALS patients should fail on tasks that involve non-monotonic reasoning in planning and language comprehension, i.e., tasks that require participants to withdraw inferences that are no longer valid, based on novel information (Stenning and van Lambalgen, 2008; Baggio et al., 2016).

Finally, our case studies suggest that abnormal responses in tasks that require comprehension of complex constructions may, in certain cases, occur in absence of aphasic symptoms. This raises the possibility that tests based on theoretical syntax and semantics are, in some patient populations, more adept than standardized neuropsychological test batteries at detecting subtle and possibly dysfunctional differences in performance. This point has been made in the past. For example, the need for novel comprehensive batteries to assess *syntactic* deficits in aphasia in the context of clinical and neuroimaging research has been emphasized before (Bastiaanse et al., 1996, 2003; Kay and Terry, 2004; Howard et al., 2010; Cho-Reyes and Thompson, 2012; Kiran et al., 2013). Specific proposals involve the use of insights from theoretical linguistics in the design of testing tools (e.g., specific tests for the number and the optionality of arguments in verb argument structure in the Northwestern Assessment of Verbs and Sentences (NAVS), by Cho-Reyes and Thompson (2012); for tests of aphasia and short-term memory deficits, which include syntactic complexity measures, (see Garraffa and Grillo, 2008; Cecchetto et al., 2012). To our knowledge, however, there is no proposal arguing for the potential methodological and clinical benefits of introducing complementary tests based on formal semantics. Yet, the range of structures investigated in semantics is sufficiently broad for tests to achieve an extensive coverage of (impaired) functions in receptive and expressive language: logical operators, quantifiers, modals and evidentials, tense and aspect, and much more (Gamut, 1991). Of particular interest are those linguistic processes that do not strictly adhere to the principle of compositionality (where the meaning of a complex expression, e.g., a phrase or sentence, can be derived from the meaning of its parts and from the syntactic mode of composition; for discussion, see Partee, 2003): complement coercion, aspectual coercion and related phenomena are examples of non-strictly-compositional operations on sentence meaning. It is conceivable that compositional semantic processes largely rest upon syntax, so that when the latter is impaired, so will be the former. Non-compositional computation, however, may rely more on knowledge-based inference (as was discussed above in relation to aspectual coercion), and therefore disorders of syntactic vs. non-compositional processing may dissociate in patients. That is one reason why tests based on logical semantics could provide new valuable information in assessing language disorders. Another reason is that traumata and pathologies of the nervous system may initially affect rather subtler and seemingly peripheral aspects of language, indeed as described by semantics, and only later impair the kind of fundamental lexical and syntactic functions assessed in standard test batteries. (The periphery-to-core decay of semantic knowledge in neurodegenerative disorders has been discussed by Rogers and McClelland, 2004). Thus, advanced tests of phrase or sentence level semantic comprehension may provide *predictors* of later impairment in basic linguistic skills, such as lexical knowledge and core syntax. The present study suggests that testing for advanced semantic capacity is possible in practice, even with patients that show severe motor dysfunction (e.g., dysphasia).

## Author contributions

GB designed and conducted research; GG, LV, and RE screened the patients and organized the testing sessions; GB, GG, LV, and RE wrote the manuscript.

### Conflict of interest statement

The authors declare that the research was conducted in the absence of any commercial or financial relationships that could be construed as a potential conflict of interest.
