# Supplementary material for: Formal Semantics in the Neurology Clinic: Atypical Understanding of Aspectual Coercion in ALS Patients
Source: Front Psychol. 2016 Nov 4;7:1733. doi: 10.3389/fpsyg.2016.01733 (PMC5095610; doi:10.3389/fpsyg.2016.01733)
Supplement: Supplementary file 1 [file DataSheet1.docx]

***Appendix: Patients’ understanding of aspectual coercion***

During testing, for a subset of the trials (~10 per patient), the experimenter asked why the patient had given a particular ‘yes’ or ’no’ response. Data from these brief dialogues provide information that is not available from analyses of binary response patterns. ALS patients are, for example, aware that the use of certain expressions, such as the temporal prepositions ‘in’ and ‘for’ (‘in’ and ‘per’ in Italian), may be disallowed, depending on the aspectual class of the VP. Knowledge of such constraints is revealed by spontaneous corrections of the grammar of sentences (capitals indicate correction-stressed words; the item number is shown in round brackets; the case number and the response provided are shown in square brackets; English translations are given in italics):

(38) Guidare l’automobile in un paio d’ore. (Additive ACT⤳ACC)

*To drive the car in a couple of hours.*

“Guidare l’automobile PER un paio d’ore.” [Case 3; negative]

*“To drive the car FOR a couple of hours.”*

(65) Andare in bicicletta in un paio d’ore. (Additive ACT⤳ACC)

*To ride the bike in a couple of hours.*

“Sarebbe PER un paio d’ore.” [Case 6; negative]

*“It should be FOR a couple of hours.”*

At the group level, in additive coercions patients show the lowest proportion of affirmative responses overall, and that is where their performance is more similar to controls and closer to chance. The expected pattern here is a decrease in the frequency of affirmative responses, reflecting the fact that ‘in’ modifiers are typically fine with accomplishments and bad with activities, while ‘for’ modifiers are usually fine with activities and bad with accomplishments. This knowledge appears to be largely available to both controls and ALS patients. The same applies to knowledge of aspectual classes. Occasionally, ALS patients reject some instances of coercion commenting their decisions with lexical (i.e., verb) replacements:

(112) Ritrovare le chiavi per un quarto d’ora. (Cross ACH⤳ACT)

*To find the keys for a quarter of an hour.*

“CERCARE le chiavi.” [Case 6; negative]

*“To LOOK FOR the keys.”*

This response reveals awareness that a ‘for’ modifier requires an activity: hence, the patient proposes to replace ‘ritrovare’ (‘to find’, ACH) with ‘cercare’ (‘to look for’, ACT).

ALS patients’ responses to the experimenter’s inquiries following rejected additive coercions indicate that basic representations of the typical duration of events, particularly of the distinction between instantaneous and temporally extended events, is largely preserved in many ALS patients:

(5) Accendere la televisione in mezz’ora. (Additive ACH⤳ACC)

*To switch on the television in half an hour.*

“Quello è istantaneo.” [Case 2; negative]

*“That’s instant.”*

“Accendere si accende subito.” [Case 5; negative]

*“It switches on immediately.”*

(68) Bucare la bicicletta in un paio d’ore. (Additive ACH⤳ACC)

*To get a flat bike tyre in a couple of hours.*

“Forse si sgonfia in tanto tempo, ma bucare no.” [Case 2; negative]

*“Perhaps it deflates in so much time, but piercing no.”*

“Si buca subito.” [Case 4; negative]

*“Piercing is immediate.”*

(140) Riporre un cacciavite in qualche minuto. (Additive ACH⤳ACC)

*To put back a screwdriver in a few minutes.*

“Forse se devi cercare dove metterlo.” [Case 2; negative]

*“Perhaps if you have to look for where to put it.”*

These comments also indicate that ALS patients can understand that turning an ACH into an ACC requires adding a process representation to the meaning of the VP: a deflation process in (68), and a search process in (140). A similar remark applies to rejected cross coercions:

(103) Scuocere la pasta per qualche minuto. (Cross ACH⤳ACT)

*To overcook pasta for a few minutes*.

“Una volta che è scotta è scotta.” [Case 1; negative]

*“Once it’s overcooked, it’s overcooked.”*

(67) Bucare la bicicletta per un paio d’ore. (Cross ACH⤳ACT)

*To get a flat bike tyre for a couple of hours.*

“Una volta che è bucata è bucata.” [Case 1; negative]

*“Once it’s pierced, it’s pierced.”*

“Improbabile, si buca subito.” [Case 6; negative]

*“Unlikely, it gets pierced immediately.”*

(130) Spezzare una matita per due minuti. (Cross ACH⤳ACT)

*To break a pencil for two minutes.*

“Una volta che è spezzata…” [Case 4; negative]

*“Once it’s broken…”*

(31) Comprare il giornale per un quarto d’ora. (Cross ACH⤳ACT)

*To buy the newspaper for a quarter of an hour.*

“Si compra e si tiene.” [Case 4; negative]

*“One buys it and keeps it.”*

“Uno compra il giornale e lo tiene.” [Case 6; negative]

*“One buys the newspaper and keeps it.”*

(112) Ritrovare le chiavi per un quarto d’ora. (Cross ACH⤳ACT)

*To find the keys for a quarter of an hour.*

“E perderle di nuovo?” [Case 12; negative]

*“And losing them again?”*

These response patterns show that ALS patients can represent the typical duration of events (e.g., piercing a tyre is instantaneous), and understand that some events cannot be sensibly iterated: e.g., one cannot break a pencil for X-time, since breaking it once prevents one from breaking it again (Case 4 to 130); one cannot buy the same newspaper for X-time, as once it is bought one keeps it (Cases 4 and 6 to 31); one cannot find the keys for X-time, unless one loses them again (Case 12 to 112).

As noted in Results, ALS patients tend to accept coercing sentences more often than controls. Their performance is more similar to controls in additive coercion (relative to the other conditions). Indeed, patients appear able to transform activities and achievements into accomplishments:

(41) Avviare l’automobile in un paio d’ore. (Additive ACH⤳ACC)

*To start the car in a couple of hours.*

“Se è guasta.” [Case 12; affirmative]

*“If it is broken.”*

This is one instance of the kind of causal reasoning that is involved in aspectual coercion: for a car-starting event (typically instantaneous) to last a couple of hours one must (a) introduce a process preceding the culmination event (e.g., a series of failed attempts at starting the car, eventually leading to a successful ignition) and (b) formulate a hypothesis as to why such a process may be necessary (e.g., the car is broken). The coerced event structure is thus linked to general knowledge of events and their causes. That is precisely the role of the *constraints on coercion* discussed in the Introduction: if linking to general knowledge (or discourse etc.) does not license the event structure that would result from aspectual coercion, then coercion is *not* applied. If the cognitive mechanisms supporting constraints on coercion are disabled, coercion is applied *despite* awareness of a missing link between the output event structure and stored knowledge and discourse information. For example (see Results), ALS patients accept a majority of subtractive coercions where controls perform at chance:

(43) Lavare l’automobile per un paio d’ore. (Subtractive ACC⤳ACT)

*To wash the car for a couple of hours.*

“Troppo.” [Case 12; affirmative]

*“Too much.”*

Here, the patient’s response suggests that he accepts the event as possible *despite* thinking ‘a couple of hours’ is an unusually long time for washing a car. The same patient endorses the null coercion counterpart of (43) (i.e., ‘Lavare l’automobile in un paio d’ore’; ‘To wash the car in a couple of hours’) without comment. Knowledge, which the patient clearly possesses, that 2 hours is too long for washing to last with no certain culmination, is not sufficient reason for this patient to block the application of coercion and reject (43).

Instances of accepted cross coercion indicate how patients manage to accommodate coerced event structures within prior knowledge of events and cause-effect relations, which would potentially block coercion. As reported in Results, these are constructions where ALS patients show higher acceptance rates than controls. Their responses to the experimenter’s queries show how elastic patients’ representations of verb meanings can be:

(49) Prendere l’autobus per dieci minuti. (Cross ACH⤳ACT)

*To take the bus for ten minutes.*

“Per una fermata.” [Case 4; affirmative]

*“For a single stop.”*

(76) Bruciare la torta per un’ora. (Cross ACH⤳ACT)

*To burn the cake for one hour.*

“Brucia la casa!” [Case 9; affirmative]

*“The house burns!”*

(4) Accendere la televisione per mezz’ora. (Cross ACH⤳ACT)

*To switch on the television for half an hour.*

“Lasciarla accesa.” [Case 10; affirmative]

*“To leave it on”.*

‘Prendere’ in (49) denotes an instantaneous event (i.e., hopping on the bus), but Case 4 here interprets it as *being* on the bus for ten minutes (or what it takes to get from one stop to the next, according to the patient’s estimate). Similarly, in (76) ‘bruciare’ is instantaneous (the sudden and usually unnoticed transition from a well cooked cake to a burned cake), but Case 9 reads it as to *let it burn* for one hour, with the possible consequence of setting the house on fire. This patient understands the meaning of ‘bruciare’, knows the effects of the protracted burning of food in the oven, but does not put this knowledge to use in rejecting (76). Finally, Case 10 takes (4) to mean that the TV is *left on* for half an hour, avoiding an easily accessible iterative reading. His own interpretation stretches the meaning of ‘accendere’: in Italian (but the same applies to the English ‘switch on’) it is an achievement denoting a punctual event. The patient does not intend his comment as a correction of (4). He accepts this sentence, and that is his account of the event structure he has constructed. The patient understands that the meaning of ‘accendere’ (which he contrasts with ‘lasciare acceso’) does not result in an iterative reading for (4), yet he does not reject (4). As with the cases discussed above, these data suggest a weakening (or anything functionally equivalent) of the constraints that should determine when aspectual coercion can be applied.
